# Anterior lumbar interbody fusion (ALIF): biometrical results and own experiences

**DOI:** 10.1007/s10143-019-01108-1

**Published:** 2019-05-20

**Authors:** Bartosz Kapustka, Grzegorz Kiwic, Paweł Chodakowski, Jan P. Miodoński, Tomasz Wysokiński, Mariusz Łączyński, Krzysztof Paruzel, Adrian Kotas, Wiesław Marcol

**Affiliations:** 1 Department of Physiology, School of Medicine in Katowice, Medical University of Silesia, ul. Medyków 4, 40-752 Katowice, Poland; 2 Department of Neurosurgery, Provincial Specialist Hospital No. 2, Jastrzębie – Zdrój, al. Jana Pawła II 7, 44-330 Jastrzębie – Zdrój, Poland

**Keywords:** Anterior lumbar interbody fusion, Degenerative disc disease, Lumbar spine surgery, Foramen measurement, Indirect decompression

## Abstract

Lumbar fusion is a mainstay in the treatment of low back pain resulting from degenerative disc disease. Anterior lumbar interbody fusion (ALIF) has become a reasonable treatment technique to achieve indirect foraminal decompression with high fusion rates. The aim of the study was to analyse the biometrical parameters of the lumbar spine and the clinical outcome. The medical records of 51 patients treated with ALIF between 2012 and 2016 were retrospectively reviewed. Anterior and posterior disc height (DH), lumbar lordosis (LL), local disc angle (LDA) and foraminal dimensions were obtained on pre- and postoperative plain radiographs and computed tomography scans using ImageJ and Surgimap software according to the pedicle–pedicle technique. To evaluate the interbody fusion status on the last follow-up CT scans, we used Bridwell criteria. Preoperative and 12 months postoperative Oswestry Disability Index (ODI) scores were determined for all patients. The average length of hospitalisation was 4 days. Most of the patients had degenerative disc disease with foraminal stenosis. Five patients had early complications like paresthesia of lower limbs, sympathetic dysfunction or wound infections, but there were no major complications. Statistically significant (*P* < .01) improvement was observed in foraminal dimensions (area = 49%, height = 33% and width = 19%), anterior DH (49%), posterior DH (69%), LDA (47%) and LL (17.5%). Posterior DH correlated significantly with foramen height improvement. Radiographic evidence of fusion according to the modified Bridwell criteria (grade I and grade II) was observed in 96% (49/51) of the patients in the last CT of the lumbar spine. We also observed significant improvement in functional recovery in 94% of patients. The mini-open ALIF approach is a reasonable alternative to the more extensive posterior approaches. ALIF significantly restores the height of the intervertebral disc, indirectly increases foraminal dimensions, increases lordosis angle with significant short and long-term pain relief and functional recovery.

## Introduction

Low back pain (LBP) is one of the most common health problems with 1-month prevalence of 23.2% [11] being also the most significant single cause of absence from work [3]. The leading cause of LBP is symptomatic intervertebral disc degeneration leading to intervertebral foramen and spinal canal stenosis.

In cases not responding to the conservative treatment, many surgical options have been established for a variety of lumbar pathologies. Lumbar fusion plays an essential role in the treatment of disabling low back and leg pain associated with continued abnormal motion in the affected segment [2]. Despite being an established procedure for a different spine pathology, there are currently no definitive studies demonstrating that any one technique is more efficacious than others. However, there are some advantages of ALIF technique. It gives a possibility to remove more disc material as a pain resource than other approaches, provides greater bone-graft contact area and allows to avoid extensive paraspinal muscle dissection. It can also increase a disc space height and a foraminal area ensuring indirect nerve root decompression. Besides, lordotic cages can restore an angle of lumbar lordosis and improve sagittal balance [12, 20, 24].

Despite the widespread use of ALIF, there are not many studies describing changes in these parameters in a clinic. In our study, we measure a degree of indirect foraminal decompression radiologically using a standardised method proposed by Rao et al. [20] with correlation to the intervertebral disc parameters and clinical outcome.

## Materials and methods

### Subjects

From January 2012 to December 2016 in our hospital, we performed a retrospective analysis of 51 consecutive patients that underwent anterior lumbar interbody fusion surgery performed by a single surgical team (G.K, K.P). Inclusion criteria included patients ≥ 18 years of age who underwent single-level stand-alone ALIF. Indications include severe lower back pain, radiculopathy and/or neurological deficit with radiological evidence of degenerative disc disease, recurrent disc herniation or foraminal stenosis, and symptoms did not resolve with conservative treatment. The exclusion criteria were previous posterior fusion, lack of 12-month radiological or clinical follow-up and two levels of ALIF. The ALIF stand-alone technique was a standard minimally invasive retroperitoneal approach with removal of the anterior longitudinal ligament, discectomy and implantation of PEEK (polyetheretherketone) interbody spacer including fixation with four locking screws SynFix-LR (DePuy Synthes) at L5-S1 single symptomatic spine level. All cages were filled with a hydroxyapatite bone substitute. The ethical review of this study was consented by the institutional review board of our hospital.

### Evaluation of clinical results

Preoperative and latest postoperative radiologic data (lumbar spine CT scans and radiographs) were reviewed. CT scans were used to obtain a standardised foramen snapshot using the pedicle-to-pedicle (P-P) technique [20]. Radiologic parameters such as DH, local disc angle (LDA) and lumbar lordosis (LL) were measured using radiographs and Surgimap software. All measurements were performed by two independent investigators, blinded to patient details.

The clinical outcomes were measured with preoperative Oswestry Disability Index (ODI) compared to the last follow-up. All patients were followed up by their physicians at the outpatient clinics for more than 12 months.

**Fig. 1:**
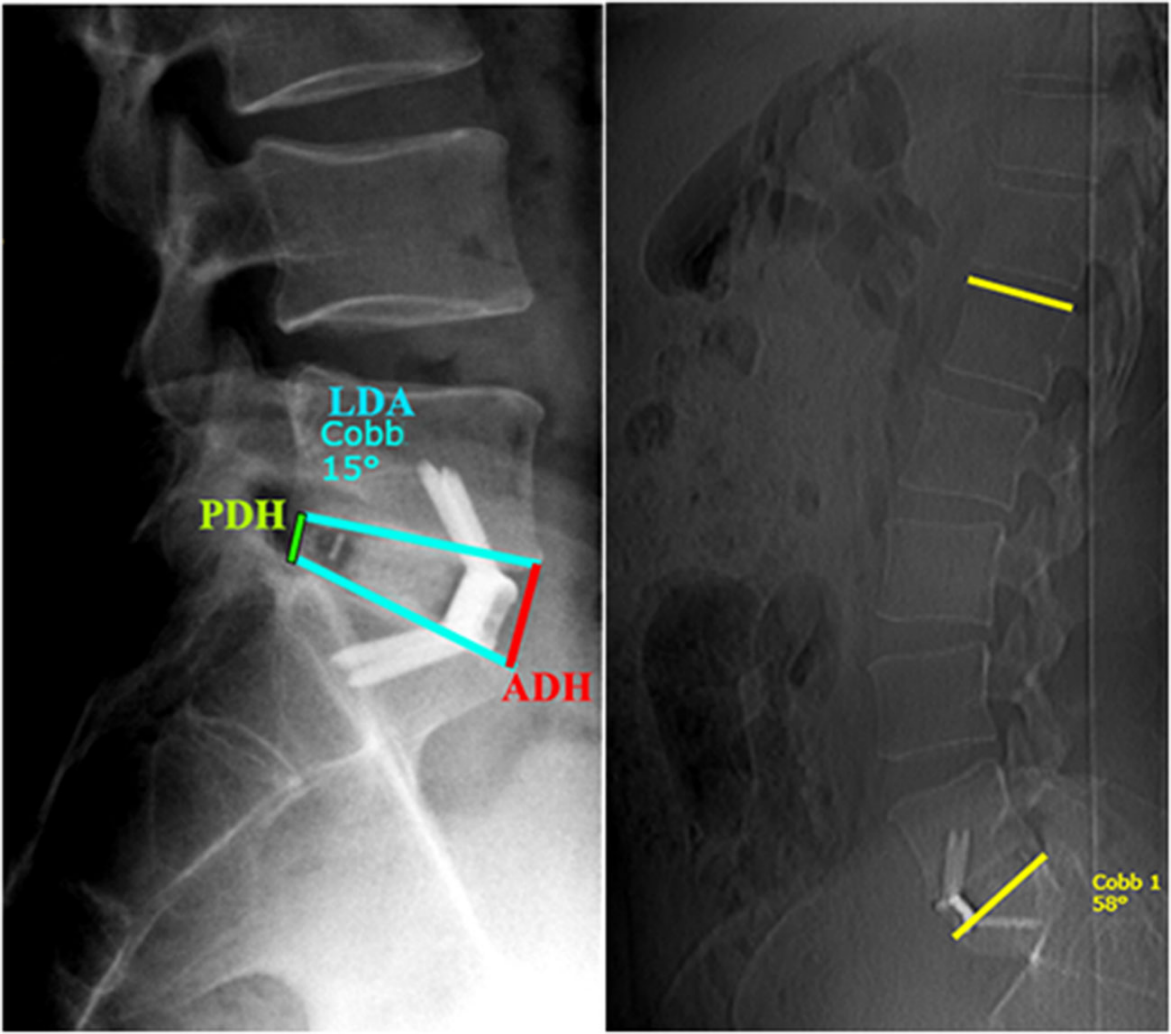
Preoperative and postoperative changes in neural foramen dimensions—foramen height (FH), foramen width (FW) and area of the foramen (FA) calculated on CT scans with P-P technique

**Fig. 2:**
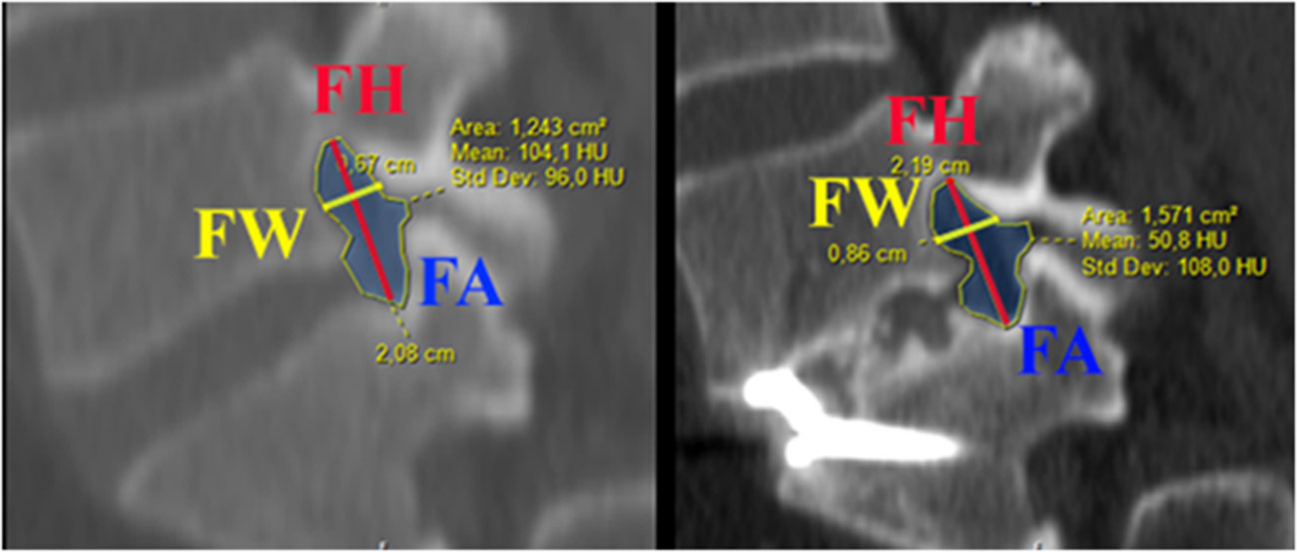
Postoperative changes in midline dimensions. On the left ADH—anterior disc height, PDH—posterior disc height, LDA—local disc angle, on the right L1-S1 lumbar lordosis obtained with Cobb method

CT scans and radiographs obtained in DICOM format were evaluated in maximum intensity projection format (MIP) using Surgimap software (Nemaris, Inc., New York, NY, USA). For the standardised analysis of the neural foramen dimensions, we used a new pedicle-to-pedicle technique (P-P technique) described by Rao et al. [20]. To calculate foraminal height (FH), width (FW) and area of the foramen (FA), we evaluated CT scans in three projections: axial plane, along with the midline of both the pedicles in the coronal plane and the sagittal plane perpendicular to the intervertebral space (Fig. 1). Lateral radiographs in standing position were used to measure disc space heights (anterior [ADH] and posterior [PDH]), lumbar L1-S1 lordosis (LL, obtained using Cobb method) and single-level lordosis (LDA) (Fig. 2). To evaluate the interbody fusion status on the last follow-up CT scans, we used Bridwell criteria (grade I—fused with remodelling and trabeculae present; grade II—graft intact, not fully remodelled and incorporated, but no lucency present; grade III—graft intact, potential lucency present at top and bottom of graft; grade IV—fusion absent with collapse/resorption of the graft).

### Statistical analysis

Differences in lumbar lordosis, local disc angle, disc heights and foramen dimensions were calculated using paired Student’s *t* tests. Paired Wilcoxon signed-rank tests were used to compare preoperative and postoperative ODI scores. In order to evaluate, the correlation of foraminal parameters and ODI Spearman’s coefficient of rank correlation was determined; to analyse the correlation between midline and foraminal parameters, Pearson’s coefficient correlation was used. The differences were considered statistically significant at *P* < 0.05. Analyses were conducted using Statistica software (StatSoft, USA).

## Results

**Fig. 3:**
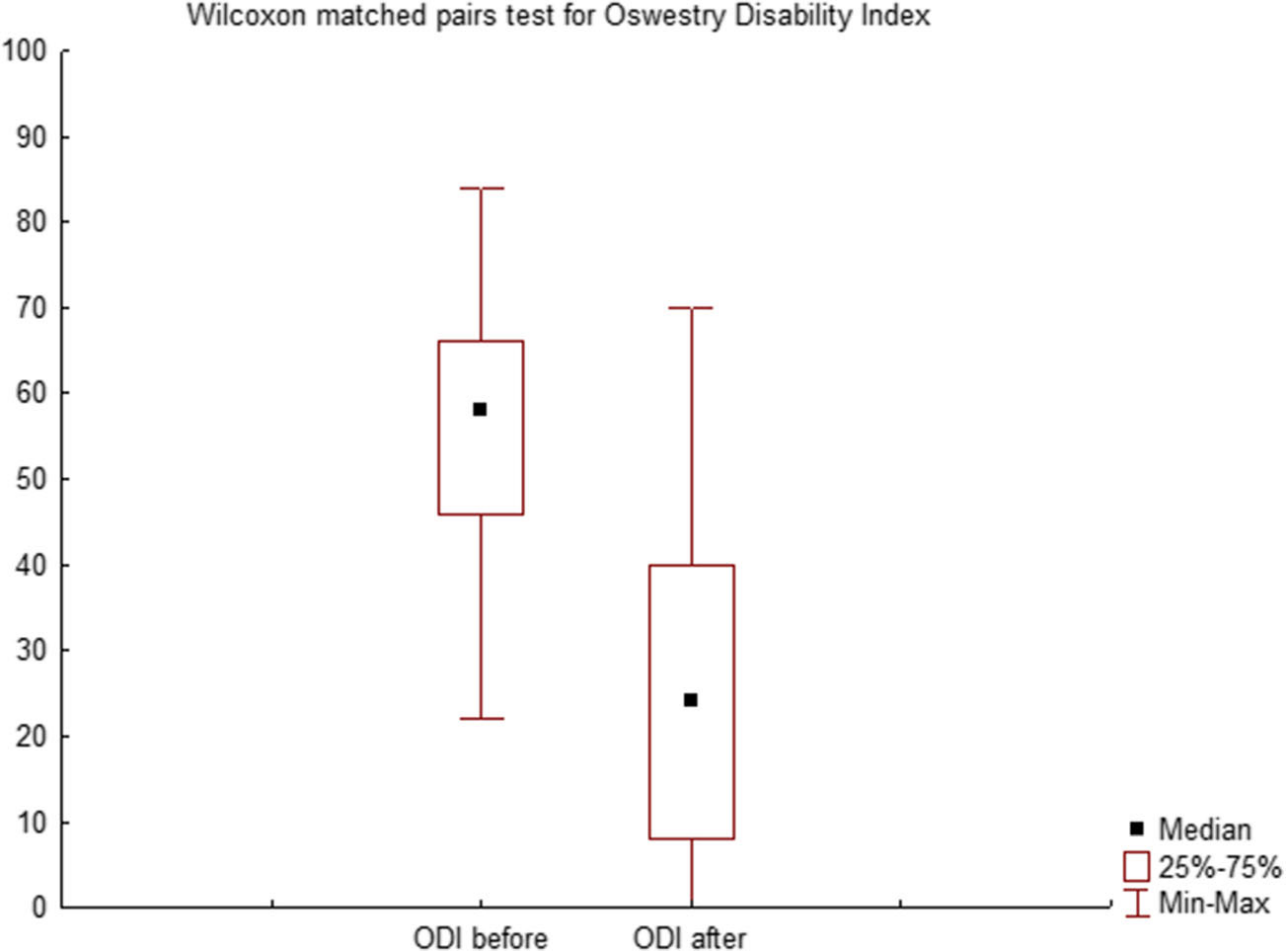
Graph showing improvements in the Oswestry Disability Index scores in patients (*P* < 0.01)

**Fig. 4:**
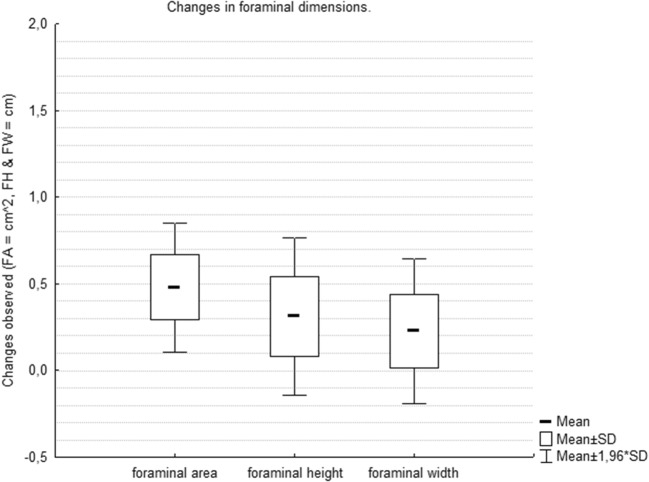
Graph showing improvements in the foraminal dimensions in patients after ALIF surgery (*P* < 0.01)

There were 51 patients with single L5-S1 levels operated. Mean age was 41.7 years (± 8.4 years, 24–59) with 27 men and 24 women. The implant sizing varied across patients in accordance with the disc height of neighbouring healthy lumbar discs, median implant size was 13.5 mm (12–15 mm) with 12° lordotic angle (48 patients vs 3 patients treated with 8° angle implant) to ensure sufficient distraction. We observed five (9.8%) early complications—two patients complained transient paresthesia of lower limbs extending throughout the L5 dermatome. One patient had sympathetic dysfunction, manifesting as left-sided leg warmth and swelling these symptoms resolved within 6 months). There were two (3.9%) cases of superficial wound infection treated with oral antibiotics, with no deep wound infections requiring reoperation or intravenous therapy. Median preoperative ODI score was 58 (22–84) and postoperatively 24 (0–70). We observed significant improvements in 94% of patients (Wilcoxon matched pair test, *P* < 0.01; Fig. 3). Postoperative follow-up for radiologic parameters was a mean of 28 months (12–59). Preoperative and postoperative foramen measurements and midline data are outlined in Tables 1 and 2. Postoperative FA grew on an average by 48.5% (right) and 49.5% (left), FH by 33.3% (right) and 21.2% (left), whereas FW improved by 19% (right) and 16.3% (left) and were statistically significant (paired Student’s *t* test, *P* < 0.01; Table 1, Fig. 4). Significant increases were seen in ADH (49.5%), PDH (69.4%) and LDA (47%), whereas a significant moderate improvement was seen in LL (17.5%) (paired Student’s *t* test, *P* < 0.01; Table 2). Of all the disc parameters, only posterior DH change was found to be a significantly correlated with FH change (Pearson’s correlation coefficient, *P* < 0.01, *R* = 0.48) but not correlated with FA or FW changes. We also observed a significant correlation between ODI score reduction and FA and FH improvement (Spearman’s correlation coefficient, *P* < 0.01, *R* = − 0.33 and − 0.43, respectively) (Table 3). Radiographic evidence of fusion according to the modified Bridwell criteria (grade I and grade II) was observed in 96% (49/51) of the patients in the last CT of the lumbar spine.

**Table 1:** Results of preoperative and postoperative foraminal dimensions analysis

Parameter	Mean (SD)	Mean (SD)	% Change	*P*
	Preoperative	Preoperative		
Right				
FA (cm^2^)	0.99 (0.35)	1.47 (0.30)	48.5	.00
FH (cm)	1.44 (0.25)	1.92 (0.24)	33.3	.00
FW (cm)	0.79 (0.14)	0.94 (0.13)	19.0	.00
Left				
FA (cm^2^)	0.97 (0.32)	1.45 (0.33)	49.5	.00
FH (cm)	1.56 (0.34)	1.89 (0.33)	21.2	.00
FW (cm)	0.80 (0.15)	0.93 (0.12)	16.3	.00

*SD* standard deviation, *FA* foraminal area, *FH* foraminal height, *FW* foraminal width

**Table 2:** Results of preoparative and postoperative midline parameters comparison

Parameter	Mean (SD)	Mean (SD)	% Change	*P*
	Preoperative	Preoperative		
ADH (cm)	1.05 (0.28)	1.57 (0.17)	49.5	.00
PDH (cm)	0.49 (0.09)	0.83 (0.17)	69.4	.00
LDA (^o^)	10.22 (3.99)	15.02 (2)	47.0	.00
LL (^o^)	44.15 (6.6)	51.86 (6.35)	17.5	.00

*ADH* anterior disc height, *PDH* posterior disc height, *LDA* local disc angle, *LL* lumber lordosis

**Table 3:** Results of Spearman correlation analysis between foraminal dimensions and Oswestry Disability Index (ODI) score

Parameters	Spearman—R	*t*(*N*−2)	*P*
FA and ODI score changes	− 0.33	− 3.07	.00
FH and ODI score changes	− 0.43	− 4.13	.00

*FA* foraminal area, *FH* foraminal height, *ODI* Oswestry Disability Index questioner

## Discussion

The ALIF procedure has been used to treat painful degenerative disc disease with or without segmental instability in the lower lumbar spine. Anterior approach allows complete resection of the degenerated disc and more or less eliminates a source of pain, at the same time leads to distract and reshape the disc space affecting the regional and local spine alignment including disc height/angle and lumbar lordosis [16, 20]. Hasegawa et al. in the cadaveric study noted intervertebral disc heights and foraminal height as a critical feature for significant nerve root compression inside the foramen. They reported the critical measures for posterior disc height equal to or less than 4 mm and 15 mm or less for foraminal height [10]. ALIF is found to be effective in many spinal disorders requiring anterior column support and fusion. Comparing to posterior spine approaches, the retroperitoneal anterior approach spares iatrogenic trauma to the paraspinal muscles, spinal nerves eliminating epidural scarring and perineurial fibrosis [15, 18, 25] and does not involve removal of posterior bony structures. In contrast to posterior or posterolateral approaches like posterior/transforaminal lumbar interbody fusion (PLIF/TLIF) familiar to most spine surgeons, ALIF restoring disc height allows reexpansion of the lateral foramen and indirect decompression of spinal nerve [18, 20]. There is a low risk of neurological damage. However, the anterior approach requires exposure and mobilisation of the great blood vessels, peritoneal contents, ureter and sympathetic plexus involving the risk of atypical in the realm of standard spine procedure complication which rate can be relatively high [22]. McDonnell et al. published a study of over 400 operated patients with 11% of major and 24% of minor complication rate related mainly to the retroperitoneal approach [17]. The venous injury is the most common, but arterial wall dissection or rupture is the most serious iatrogenic injury; therefore, careful preoperative evaluation of vessels on magnetic resonance imaging (MRI) or abdominal computed tomography angiography (CTA), meticulous preparation and manipulation of an iliolumbar vascular complex is required. Being aware of complications of the procedure, however, ALIF has been widely accepted for various lumbar pathologies. Rao et al. in a prospective study described ALIF as an effective both clinically and radiologically treatment for patients presenting with different spinal pathologies reporting statistically significant improvement in pain, functional and well-being measures in degenerative disc disease, spondylolisthesis, pseudarthrosis or spinal deformity. The study confirmed ALIF as the promising procedure for degeneration, instability and even deformity of the lumbar spine [19–21]. However, some of the anatomical details of a degenerative spine as greater degrees of spondylolisthesis and severe facet arthropathy can make anterior indirect decompression insufficient and influence the failure of ALIF resulting in persistent foraminal stenosis and an unfavourable outcome [7]. Severe arthropathy that limits capacity to restore foraminal height and superior articular process impingement on neural foramen is associated with the incomplete restoration of foraminal size despite disc distraction [7]. Sang-Ha Shin et al. in their clinical study recommended direct removal of the lesion using a microsurgical technique for anterior foraminal decompression (AFD). For this technique, approximate identification of intervertebral foramen after removal of posterior annulus fibrosus or even posterior longitudinal ligament (PLL) is mandatory; therefore, one can consider AFD as difficult and risky manoeuvre [24]. An obstacle in performing this procedure is pathology at the L5/S1 level with a steep inclination of disc space, obesity and severe facet arthropathy [7]. To overcome the mentioned above challenges nowadays, we can additionally implement spinal endoscopy. Moreover, the assistance of different spinal endoscopic systems and approaches effectively expose foraminal region and remove a fragment of the herniated disc or osteophyte as well without inadvisable bony destruction [1]. The spine companies make constant technological progression and design new implants with a stable anchoring system. Therefore, ALIF as a stand-alone procedure raises less doubt regarding insufficient segmental stabilisation and cage subsidence problem influencing long-term intervertebral foraminal dimension [21]. The crucial factors determining the insufficient tolerability of axial rotation or extension and unexpected cage subsidence besides well-designed device are appropriate bony end-plate preparation and adequate bone quality e.g. osteoporotic bone [5, 14]. Current reports showing 94–97% of fusion success rate for mini-open stand-alone ALIF surgery which is relatively superior to PLIF or TLIF procedure demonstrated that ALIF could be an effective surgical modality of interbody fusion to obtain the reliable clinical results [9, 19]. Despite the growing prevalence of anterior technique with satisfactory interbody arthrodesis, there are currently no definitive studies demonstrating that any one technique is more efficient than the other in terms of fusion rate or clinical outcomes [18]. Our results of single-level stand-alone L5-S1 ALIF procedure try to supplement recent literature demonstrating the problem of both the segmental stabilisation and segmental foraminal stenosis [20, 26]. For our retrospective study, we implemented the new pedicle-to-pedicle (P-P) technique described by Rao et al. [20]. In our study, we used pre- and postoperative three-dimensional CT scans to analyse foraminal parameters similarly to Rao et al. [20]. They described normal values as FA from 1.25 to 2.25 cm^2^, FH from 11 to 19 mm and FW from 5 to 12 mm [20]. The foraminal area on preoperative CT observed in our study was lower than normal values, but similar to the other studies analysing symptomatic degenerative disc disease population [6, 13, 20, 24]. Foraminal area improvement by 49% is similar to that presented by Cho et al. [6] which is 40% and Shin et al. which is 42.3% L5-S1 (but with direct anterior foraminal decompression) [24] and lower than 87% observed by Rao et al. (for L5-S1 level) [20]. It must be noted that Rao et al. reported a similar cohort of patients with L5-S1 ALIF, but in contrast to the present study, results were measured on L2-L3, L3-L4, L4-L5 and L5-S1 levels, so we were not able to compare pre- and postoperative L5-S1 foraminal dimensions [20]. Compared to Rao et al., we observed almost the same preoperative FH and FW (FH 1.44 cm and 1.56 cm vs 1.4 cm and FW 0.79 and 0.8 cm vs 0.8 cm), but in our population, higher increase in foraminal height (mean 27.3% vs 32.6% in Rao et al. study) and lower increase in foraminal width were observed (mean 17.7% vs 48.7% in Rao et al. study) [20]. Shin et al. presented a similar 25–38.2% FH improvement and a reduction of FW by (13.4–22.1%), but with direct foraminal anterior decompression [24]. We also observed lumbar lordosis, posterior and anterior DH improvement comparable to Hseih et al. study [12]. Dennis et al. observed similar 49% increase in DH [8]. Schlegel et al. reported on a cadaveric specimens that an anterior disc distraction of 10 mm caused 40% FA improvement; in our study, a mean cage height was 12.31 mm and caused proportional 49% FA increase [23], but Rao et al. study showed that 66.7% FA increase was achieved with only 7-mm disc distraction [20]. Similar to Rao et al. and Chen et al., we found a significant correlation between PDH increase and FH restoration only [4, 20]. Moreover, in a regression analysis, we observed a significant correlation between ODI questionnaire score reduction and FH and FA improvement in a follow-up. The results of our study demonstrated a positive impact of changed spinal parameters on clinical outcome. Oswestry Disability Index (ODI) score improvement was observed in 94% of our patients with a low complication rate. Two patients have complained of slight and transitional paresthesias, specific for L5 nerve root irritation, and one patient had sympathetic dysfunction. There were two (3.9%) cases of superficial wound infection. We were able to avoid any significant vascular injury, retrograde ejaculation, urological complications or abdominal muscle damage mainly thanks to appropriate patient selection and presence of well-experienced and trained access surgeon.

### Limitations

The clinical outcomes were determined during a relatively short follow-up period. Therefore, we are not able to answer the question if subsidence following stand-alone cages had a potential to affect spinal measuring, long-term clinical success and complication rate. The limited number of patients confined to L5/S1 pathology, relatively short follow-up, retrospective and nonrandomized nature are flaws of our study.

## Conclusions

Our study indicates that ALIF procedure using stand-alone cage restores foraminal dimensions, disc height, local disc angle and lumbar lordosis allowing decompression of nerve roots based on the P-P technique.
